# MagIC beads for scarce macromolecules

**DOI:** 10.7554/eLife.105335

**Published:** 2025-01-20

**Authors:** Carlos Moreno-Yruela, Beat Fierz

**Affiliations:** 1 Laboratory of Biophysical Chemistry of Macromolecules, Institute of Chemical Sciences and Engineering (ISIC), School of Basic Sciences, École Polytechnique Fédérale de Lausanne (EPFL) Lausanne Switzerland

**Keywords:** cryogenic electron microscopy, macromolecules, SpyTag/SpyCatcher, magnetic beads, chromatosome, *Xenopus*

## Abstract

Specialized magnetic beads that bind target proteins to a cryogenic electron microscopy grid make it possible to study the structure of protein complexes from dilute samples.

**Related research article** Arimura Y, Konishi HA, Funabiki H. 2024. MagIC-Cryo-EM: Structural determination on magnetic beads for scarce macromolecules in heterogeneous samples. *eLife* **13**:RP103486. doi: 10.7554/eLife.103486.

Cryogenic electron microscopy (or cryo-EM for short) has revolutionized our understanding of biology. The technique – which uses a beam of electrons fired at frozen samples to work out their structure – has revealed the three-dimensional arrangements of many large complexes that drive essential biological processes such as chromatin regulation (Nogales and Mahamid, 2024).

To prepare samples for this type of microscopy, high quality biological materials, such as protein complexes, undergo multiple stages of purification and concentration before being applied to a metal mesh grid in a thin layer. The grid is then rapidly frozen at cryogenic temperatures (Frank, 2017). High concentrations of the target macromolecules are needed to properly analyze the sample, yet most of these macromolecules are lost during the application to the grid, limiting the technique to samples with abundant molecules. To broaden the reach of cryo-EM – especially for complexes that are challenging to purify or concentrate – further advancements in simplifying the handling of a sample and minimizing the amounts of sample needed are essential.

Several strategies have been implemented to expand the scope of samples that can be used for cryo-EM. For example, analyzing samples directly after minimal purification has enabled researchers to characterize larger – albeit more abundant – assemblies, such as the proteasome, an enzyme complex consisting of multiple subunits (Kastritis et al., 2017; Verbeke et al., 2018; Ho et al., 2021). Editing a gene in one component of a macromolecular complex so that it contains the sequence for an affinity tag has further allowed researchers to isolate complexes with minimal processing. These tags can be captured directly from cell extracts using specific molecules or ‘handles’, allowing the tagged protein and the rest of the macromolecular complex to be separated more easily (Herbst et al., 2021).

Similarly, cryo-EM grids containing ‘handles’ such as Ni-NTA, streptavidin or the SpyTag/SpyCatcher system can attract the desired molecules to the grid, thereby lowering the required concentration of a sample (Wang et al., 2008; Kelly et al., 2008; Han et al., 2016; Wang et al., 2020). However, no general solution exists to date that can bypass completely the standard steps of sample purification and concentration.

Now, in eLife, Yasuhiro Arimura, Hide Konishi and Hironori Funabiki of The Rockefeller University report on the development of custom magnetic beads (known as MagIC-cryo-EM beads) to separate low concentration assemblies from a complex mixture and apply them directly to cryo-EM (Arimura et al., 2024). With this technique, the team were able to resolve the structure of a fundamental unit of chromatin (known as a chromatosome) formed inthe egg extract of the African clawed frog, despite this complex only being present at very dilute concentrations.

The chromatosome complex was purified from frog egg extracts using the MagIC-cryo-EM beads. The researchers then used a strong magnet to attract the magnetic beads to the cryo-EM grid membrane, which enabled them to generate sufficient cryo-EM data from a dilute sample. The MagIC-cryo-EM beads further included a protein spacer to distance the sample from the magnetic beads and to avoid interferences from the metallic core during imaging (Figure 1).

**Figure 1.**
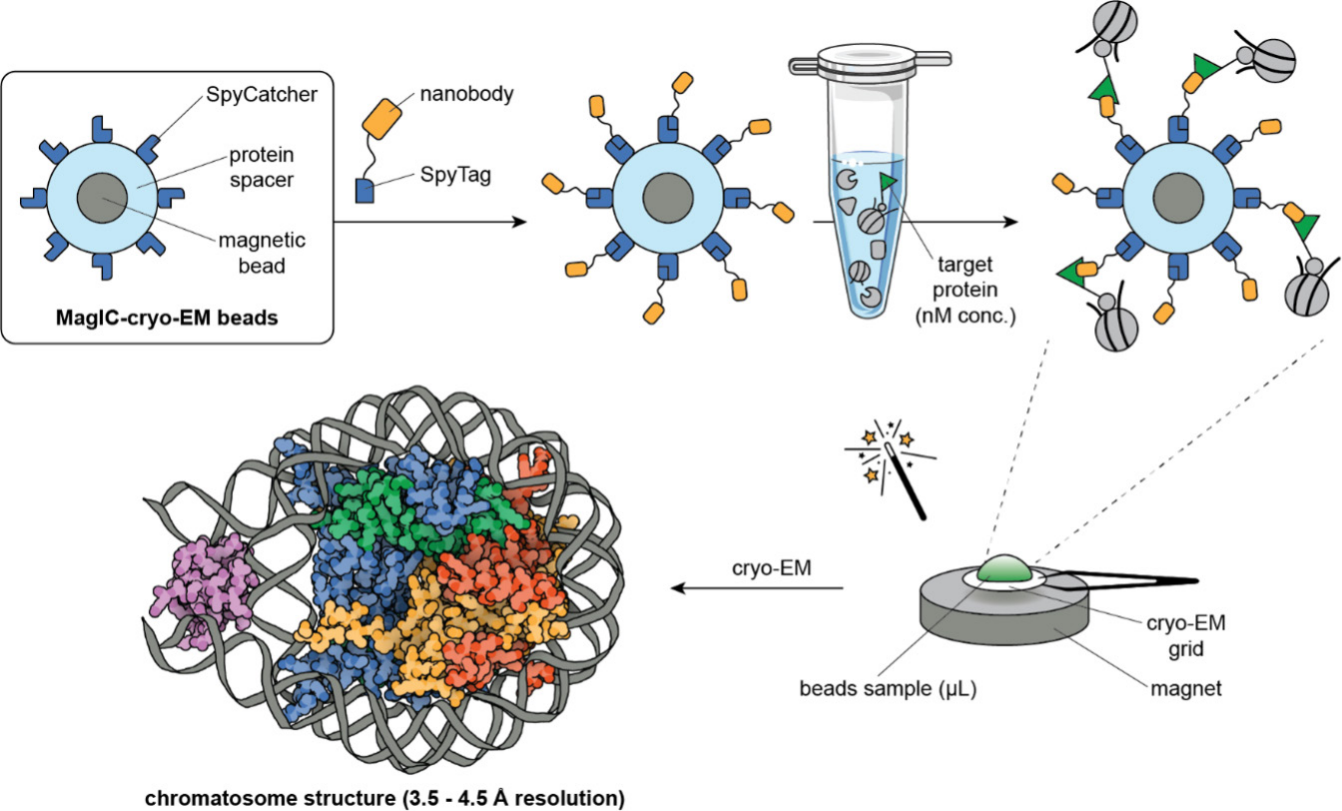
High-resolution structural imaging using customised magnetic beads. Magnetic Isolation and Concentration-cryo-EM (MagIC-cryo-EM) beads (top left box) consist of a magnetic bead (gray circle) coated with a protein spacer (light blue ring), which has SpyCatcher proteins attached to it (dark blue shapes). To isolate target proteins from a sample, a SpyTag peptide attached to a nanobody (yellow shape) that binds specifically to the target protein tag (green triangle) is added to the MagIC-cryo-EM beads using SpyCatcher/SpyTag technology. This allows the beads to capture the target proteins – and any stable complexes they are a part of – directly from cell extracts. A magnet helps to pull the beads and their cargo onto cryogenic electron microscopy (cryo-EM) grids (bottom right), which are then imaged directly, bypassing the standard intermediate purification and concentration steps. Thanks to the protein spacer, the enriched complexes can be characterized accurately without interference from the magnetic beads. Arimura et al. used this technique to reveal the chromatosome structure of the African clawed frog (bottom left, H1: purple, H2A: yellow, H2B: red, H3: blue, H4: green, DNA: grey). Chromatosome model represents PDB 4QLC (Zhou et al., 2015). Microtube adapted from Bioicons.com (CC BY 4.0).

The MagIC-cryo-EM beads have a protein on their surface known as 'SpyCatcher', which irreversibly binds to its corresponding 'SpyTag' peptide. In the system designed by Arimura et al., the SpyTag peptide is bound to a nanobody that specifically binds to a tag in the complex of interest. This allowed the chromatosome complex within frog egg extracts to bind to the MagIC-cryo-EM beads.

Cryo-EM showed that the chromatosome contained fragments of DNA wrapped around a core of histone proteins (H2A, H2B, H3 and H4) that were further bound by the linker histone H1.8. The resulting high-resolution structures also highlighted that, contrary to a previous hypothesis, there were no positional differences of H1.8 on the chromatosome between the interphase and metaphase of a cell cycle.

Arimura et al. also devised a new protocol, called Duplicated Selection to Exclude Rubbish particles (or DuSTER for short), which helps identify useful cryo-EM signals and eliminate cell debris. This provided, for the first time, structural evidence that a group of proteins that support protein folding and are known as chaperones (in particular NPM2) were involved in binding the H1.8 dissociated from the DNA. The distinct conformational states during the different phases of the cell cycle could lead to potential changes in the binding affinities of H1.8 for NPM2.

The new cryo-EM protocol designed by Arimura et al. makes it possible to study macromolecular complexes extracted directly from cell extracts at low concentrations, eliminating the need for intermediate fractionation, purification or concentration steps. In addition, the modular design of the MagIC-cryo-EM beads allows the tool to be used with a range of nanobodies to target different tags or even native proteins. Together, these methods hold promise towards a broad application and constitute a valuable resource that will help to illuminate the nanoscopic organization of life.
